# Dural Follicular Non-Hodgkin Lymphoma Mimicking a Chronic Subdural Hematoma: A Case Report and Literature Review

**DOI:** 10.7759/cureus.115817

**Published:** 2026-09-05

**Authors:** Carlos A Arellanes-Chavez, Ernesto Ramos-Martínez

**Affiliations:** 1 Neurosurgery, Autonomous University of Chihuahua, Chihuahua, MEX; 2 Pathology and Laboratory Medicine, Patologia E Inmunohistoquimica de Chihuahua SC, Chihuahua, MEX

**Keywords:** chronic subdural hematoma (csdh), differential diagnosis, dural enhancement, primary dural lymphoma, rosai-dorfman disease

## Abstract

Primary dural lymphomas are rare neoplasms that can clinically and radiologically mimic a chronic subdural hematoma, delaying accurate diagnosis; the follicular subtype is an exceptionally rare variant within this group. We report the case of a 77-year-old woman with a history of arterial hypertension who, after two ground-level falls, developed progressive right-sided hemiparesis, ipsilateral hemidysesthesia, recent memory impairment, and a left hemicranial headache. Computed tomography showed a biconvex, hypodense left fronto-parietal lesion measuring 6 × 3 cm, and magnetic resonance imaging demonstrated a T2-hyperintense lesion with dural enhancement after contrast administration, findings initially interpreted as a probable traumatic subdural hematoma versus an arachnoid cyst. Once her anemia had been corrected and she was clinically stable, she underwent craniotomy, during which the dura mater was found to be markedly thickened; histopathological and immunohistochemical studies confirmed a follicular non-Hodgkin lymphoma with an interfollicular pattern in 95% and a follicular pattern in 5%, World Health Organization grade I, CD20-positive, Ki-67 30%. The postoperative course was favorable, with improvement of the hemiparesis; she was discharged and referred to hematology, where she received chemotherapy, but died two months later. A literature review identified 14 previously reported cases of dural lymphoma mimicking a subdural hematoma, to which the present case is added. Clinical, imaging, and histopathological features suggestive of this entity are discussed, along with the main differential diagnoses of dural enhancement, including Rosai-Dorfman disease and metastatic Hodgkin lymphoma. Dural lymphoma, particularly its follicular variant, should be considered in the differential diagnosis of extra-axial lesions resembling a subdural hematoma, especially in elderly patients without a clear history of trauma or with imaging findings discordant with the expected clinical course.

## Introduction

Chronic subdural hematoma (CSDH) is one of the most common extra-axial lesions encountered in neurosurgical practice, particularly among elderly patients, and is usually diagnosed without difficulty by computed tomography (CT) and magnetic resonance imaging (MRI) [1]. However, a small but clinically important group of lesions can radiologically mimic a subdural hematoma without truly being one; primary dural lymphomas are among them.

Primary central nervous system lymphomas account for 1-7% of all non-Hodgkin lymphomas and 2-4% of all primary brain tumors, with a higher incidence among immunocompromised patients [2]. Primary involvement of the dura mater is even more infrequent and most often corresponds histologically to marginal zone lymphomas of mucosa-associated lymphoid tissue (MALT), followed in frequency by diffuse large B-cell lymphomas; dural follicular lymphoma, as documented in the present case, has been described only exceptionally in the literature [3].

Because the dura mater is physiologically devoid of organized lymphoid tissue, the pathogenesis of primary dural lymphoma remains uncertain, with a reactive or inflammatory acquisition of meninges-associated lymphoid tissue having been proposed [4]. Clinically and radiologically, these lesions can be mistaken for a CSDH, an arachnoid cyst, or a meningioma, delaying the definitive histopathological diagnosis and, consequently, timely treatment [5].

We present the case of a patient with a dural follicular non-Hodgkin lymphoma that clinically and radiologically mimicked a traumatic subdural hematoma, together with an extensive review of the literature on similar cases.

## Case presentation

History of present illness

A 77-year-old woman with a history of arterial hypertension as her only relevant comorbidity presented one month before admission with two ground-level falls, without evident head trauma and without loss of consciousness. Two weeks later, she developed progressive weakness of the right side of the body, accompanied by ipsilateral hemidysesthesia and impairment of recent memory. On admission, she reported a left hemicranial headache of 6/10 intensity on the visual analog scale, described as constant and pressure-like, accompanied by nausea and vomiting.

Physical examination

The patient had an ectomorphic build, appeared in no acute distress apart from a pained facial expression, showed no abnormal movements, and had pale integument.

Neurological examination

She was conscious and oriented in all three spheres, with no cranial nerve deficits and a normal fundoscopic examination. Right facio-corporal hemiparesis (4/5), hyperreflexia of the right hemibody, a positive Babinski sign, and associated pyramidal signs in the right lower limb were documented, without meningeal irritation or cerebellar signs.

Laboratory findings

Laboratory studies showed leukopenia (white blood cell count 3.36 ×10³/µL; reference range, 4.5-11.0 ×10³/µL) with neutropenia (absolute neutrophil count 1.20 ×10³/µL; reference range, 1.8-7.7 ×10³/µL), as well as severe anemia (hemoglobin 6.70 g/dL; reference range, 12.0-15.5 g/dL, and hematocrit 20.3%; reference range, 36%-46%).

Imaging studies

**Figure 1:**
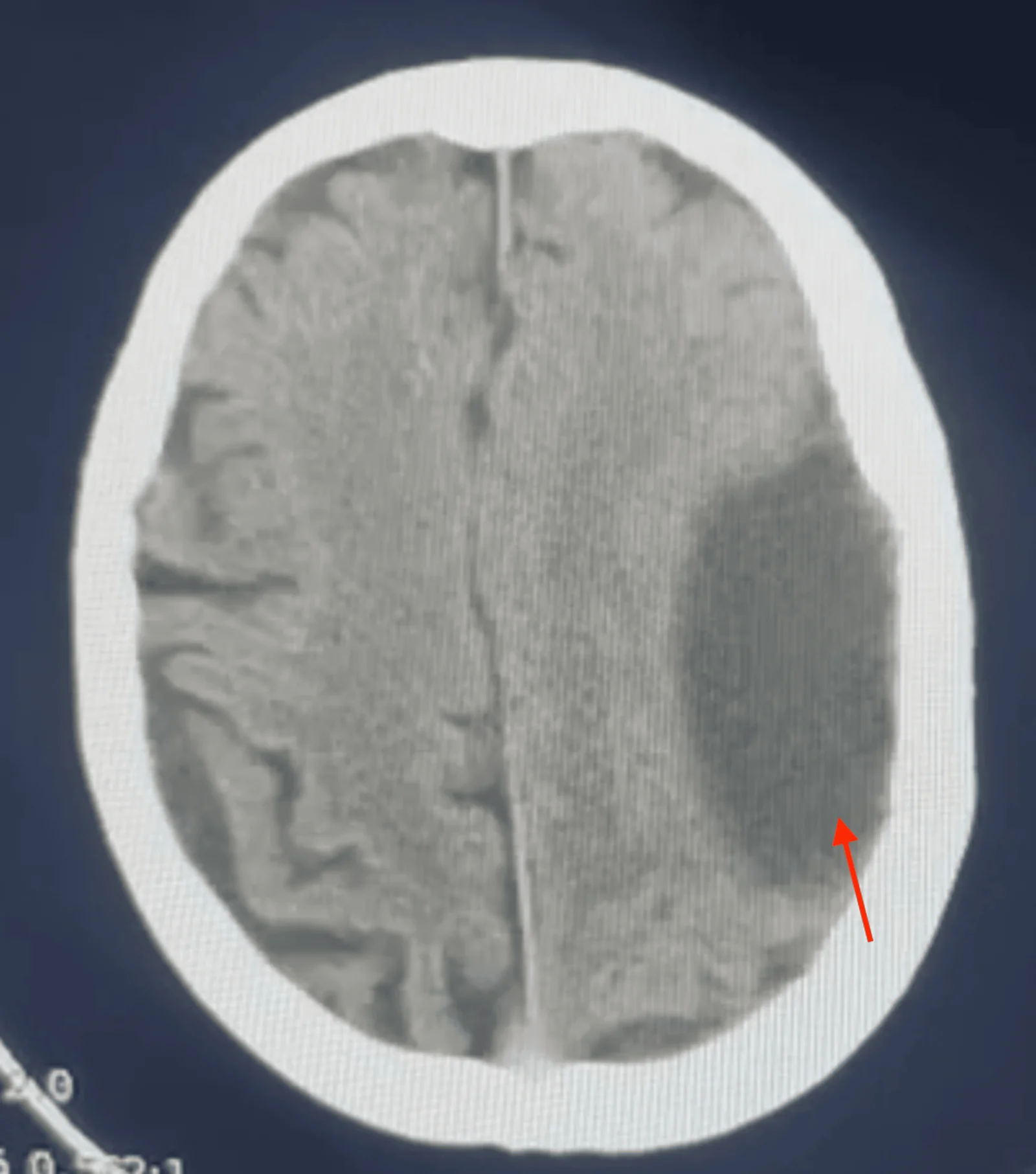
Non-contrast axial cranial CT scan showing a hypodense lesion in the left fronto-parietal region (red arrow).

Non-contrast head CT showed a biconvex, hypodense left fronto-parietal lesion measuring 6 × 3 cm (Figure 1).

**Figure 2:**
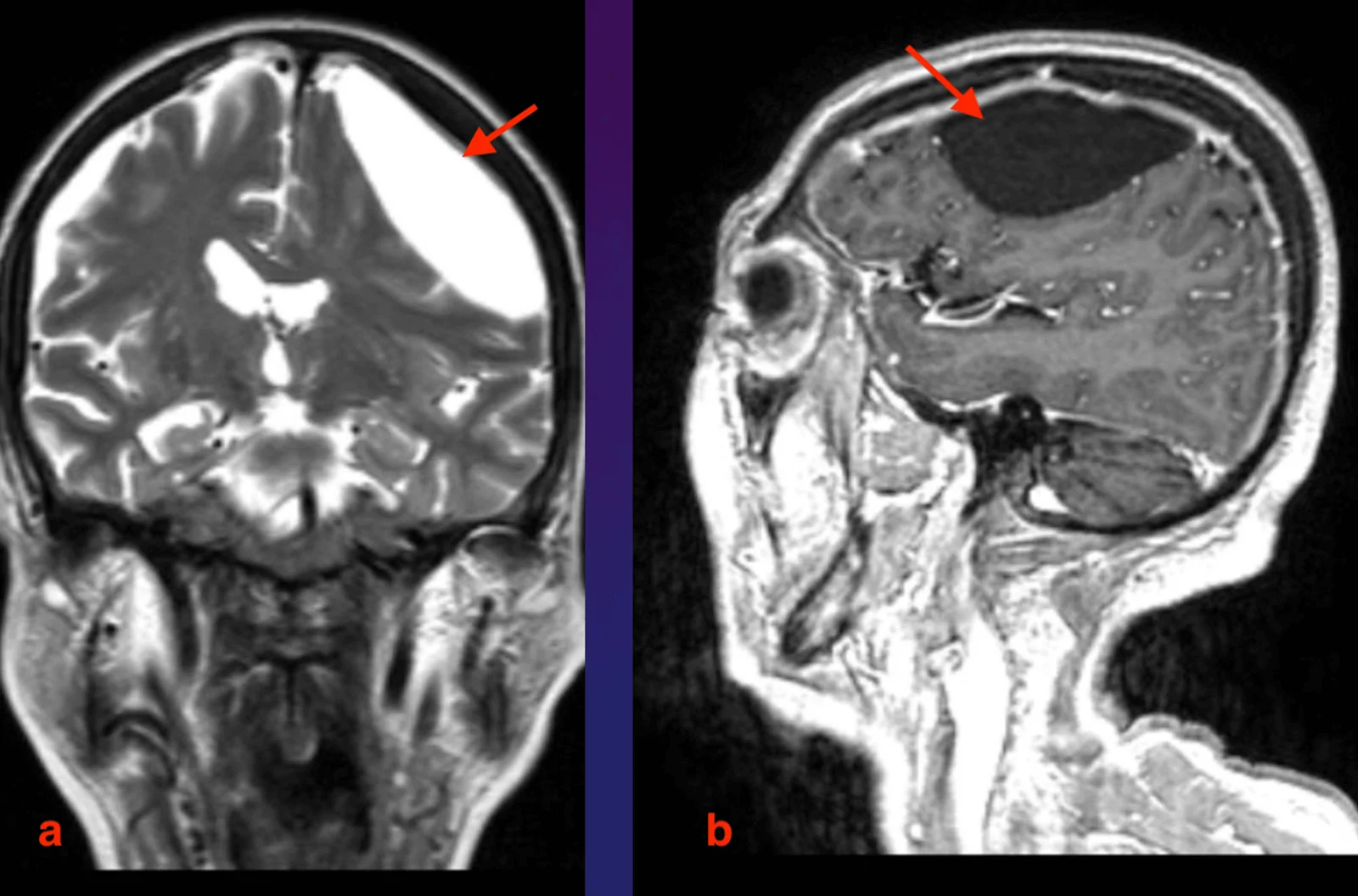
Cranial MRI: (a) Coronal T2-weighted view showing a left frontal hyperintense cystic lesion (red arrow) with displacement of the brain and the ipsilateral lateral ventricle. (b) Sagittal view with contrast medium showing dural enhancement in addition to the hypointense cystic lesion (red arrow).

Non-contrast and contrast-enhanced MRI showed, on coronal T2-weighted images, a hyperintense left fronto-parietal lesion partially displacing the ipsilateral lateral ventricle; after gadolinium administration, the sagittal sequence showed a hypointense fronto-parietal lesion with adjacent dural enhancement (Figure 2).

Based on these findings, a syndromic diagnosis of intracranial hypertension and right pyramidal syndrome was established, secondary to a probable traumatic subdural hematoma versus an arachnoid cyst.

Surgical management

**Figure 3:**
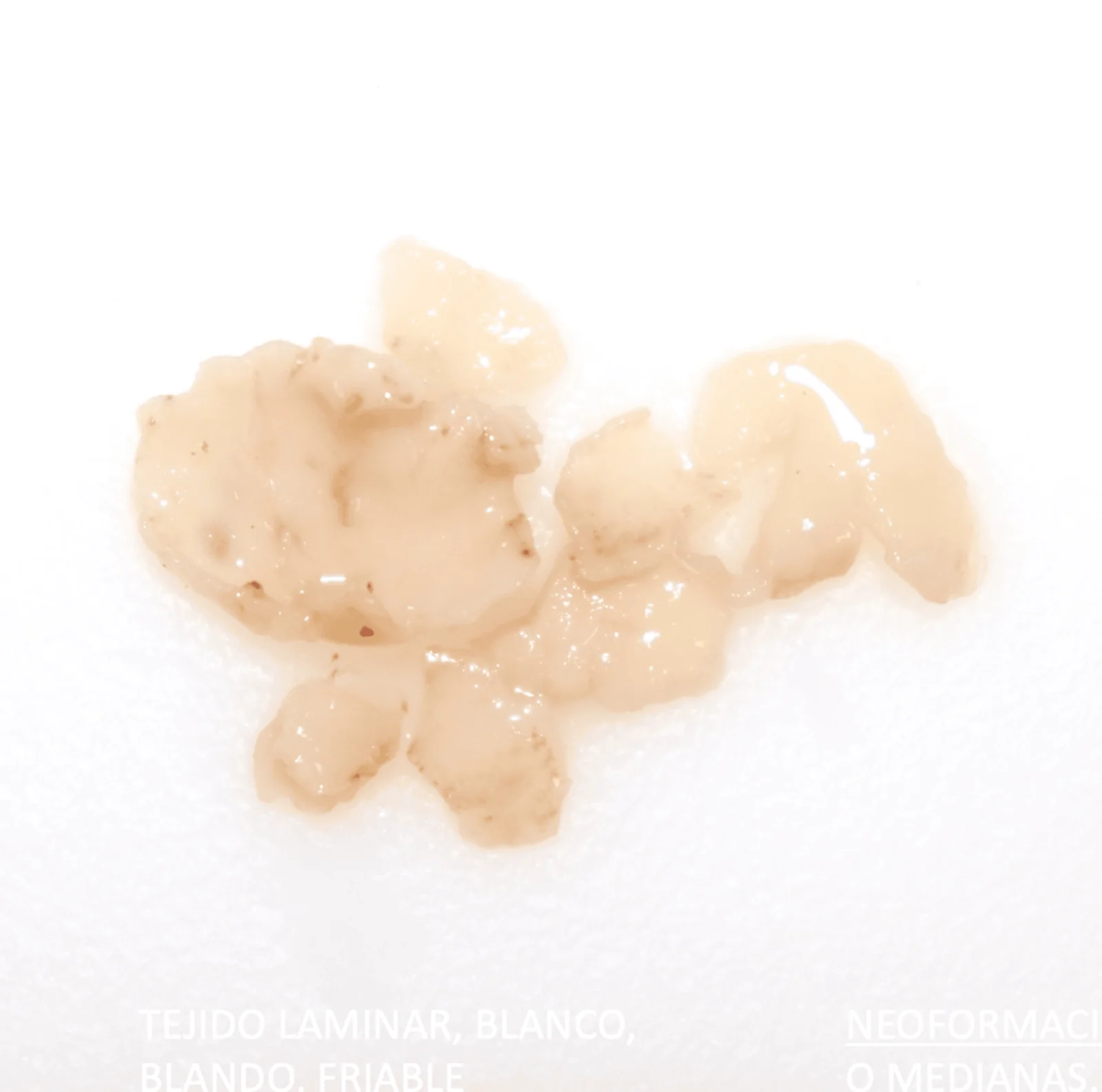
Dura mater fragments: laminar, white, soft, friable tissue.

Once the patient's anemia had been corrected and she was clinically stable, a left fronto-parietal craniotomy was performed with drainage of the cystic-appearing lesion. Intraoperatively, the dura mater was markedly thickened, and a partial resection was performed and sent for histopathological study (Figure 3).

Histopathological findings

**Figure 4:**
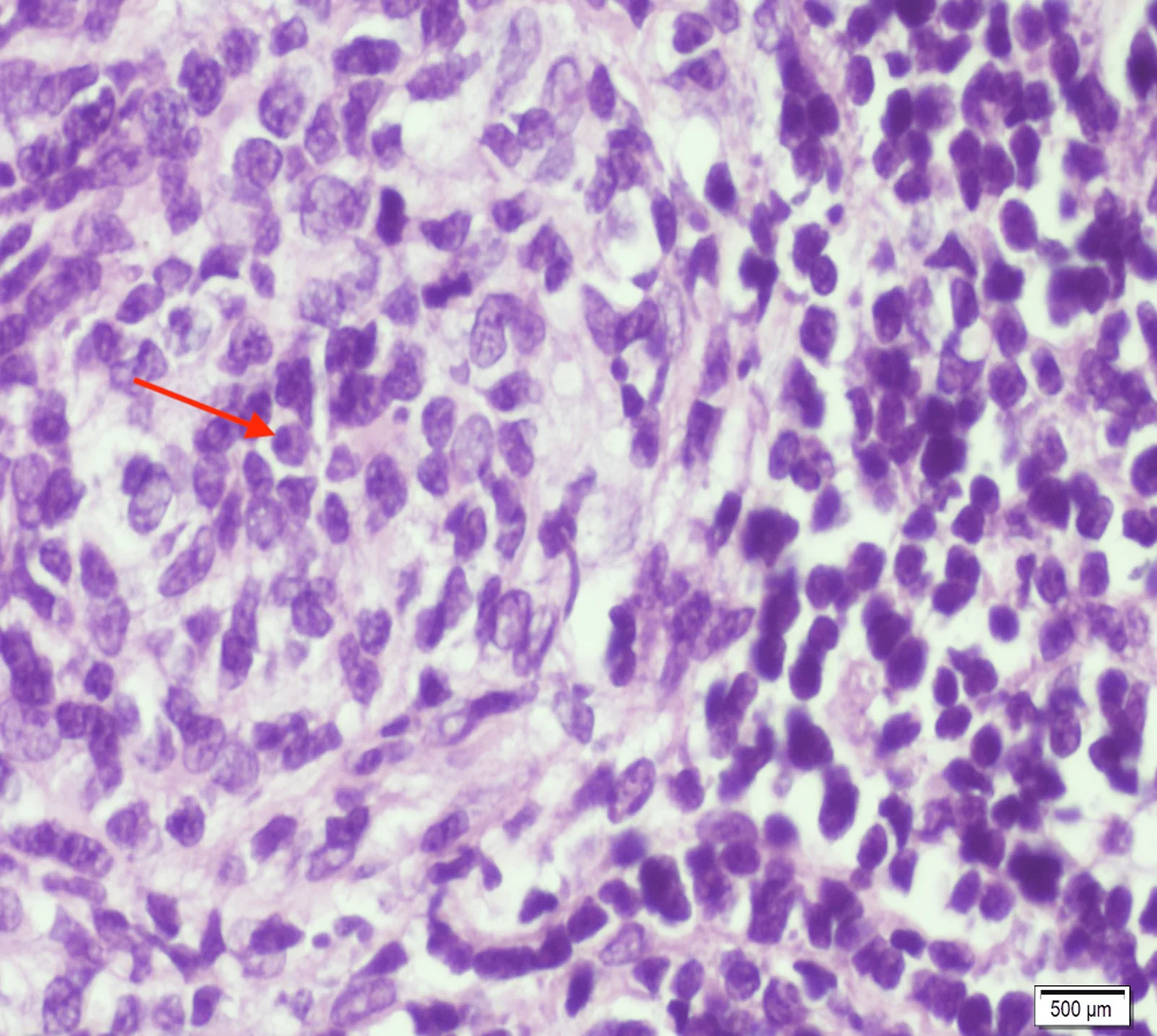
Hematoxylin-eosin slide. Neoplasm composed of small- to medium-sized cells with round, hyperchromatic nuclei and fine granular chromatin (red arrow), showing 21 mitoses per 10 high-power fields.

Microscopic examination revealed a neoplastic proliferation composed of small to medium-sized cells with round, hyperchromatic nuclei and finely granular chromatin, with 21 mitoses per 10 high-power fields (Figure 4).

**Figure 5:**
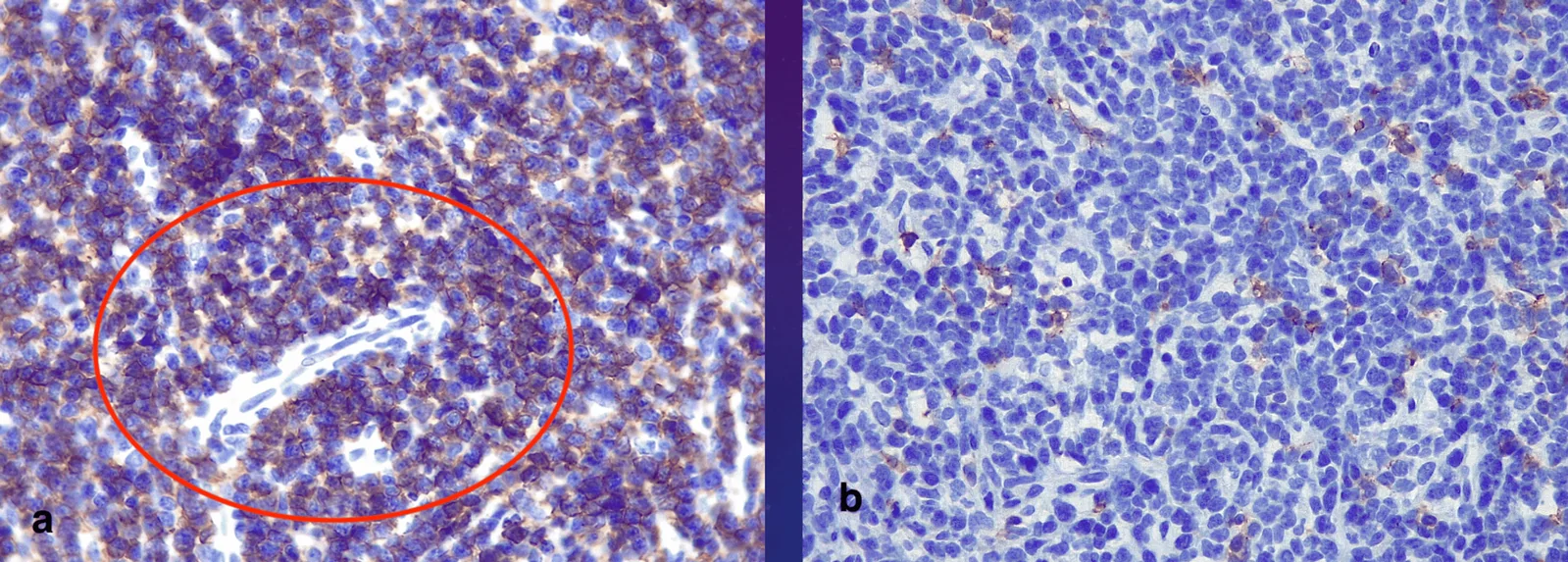
Immunohistochemistry: (a) CD20: follicular lesion (red circle) with 95% interfollicular pattern, grade I. (b). Ki-67: 30% intensity, 100%.

Immunohistochemistry for CD20 demonstrated a follicular lesion with a predominantly interfollicular pattern (95%) and a follicular pattern (5%), grade I; the Ki-67 proliferation index was 30%, with 100% intensity. These findings established the definitive diagnosis of follicular non-Hodgkin lymphoma, with an interfollicular pattern in 95% and a follicular pattern in 5%, World Health Organization (WHO) grade I (Figure 5).

Outcome

The postoperative course was favorable, with progressive improvement of the right hemiparesis. The patient was discharged and referred to the hematology department, where she received chemotherapy; however, she died two months later.

## Discussion

CSDH is the leading diagnosis when a biconvex or crescent-shaped extra-axial collection is found in an elderly patient, especially with a history of falls, as in the present case. However, the absence of a clear head trauma history, a subacute course with progressive neurological deficit, and dural enhancement after contrast administration should raise suspicion of a non-hemorrhagic lesion, including primary dural lymphoma.

Since 2000, approximately a dozen cases of dural lymphoma with a radiological presentation indistinguishable from CSDH have been reported [6], most of B-cell lineage, predominantly of the marginal zone (MALT) subtype, followed by diffuse large B-cell lymphomas and, anecdotally, Burkitt lymphoma and lymphoblastic lymphoma. Dural follicular lymphoma, as documented in this patient, is an extraordinarily rare variant; to the extent the literature review allows, only the case described by Low and Allen in 2006 corresponds to a grade I follicular lymphoma with dural involvement, although in that case the radiological presentation mimicked a meningioma rather than a subdural hematoma, and was accompanied by concomitant bone marrow infiltration [3].

Table 1 summarizes the reviewed published cases of dural lymphoma mimicking a subdural hematoma, including the case reported in this article. Most patients are elderly women (mean age over 60 years), without a clear history of trauma, with lesions predominantly fronto-parietal or fronto-temporal in location, without a clear side predominance. Prognosis varies according to histological subtype: low-grade lymphomas (MALT, follicular) generally show a favorable course after biopsy, surgery, and/or radiotherapy, whereas high-grade subtypes (diffuse large B-cell lymphoma, Burkitt lymphoma, lymphoblastic) are associated with a worse prognosis, including early recurrence or death, as observed in the cases reported by Han et al. [6] and Ramnarayan et al. [7]. Notably, the present case illustrates that a favorable immediate postoperative course and a low-grade histology do not necessarily predict a favorable long-term outcome: despite an uneventful surgical recovery and referral for adjuvant chemotherapy, the patient died two months after discharge, underscoring that systemic disease burden, advanced age, and comorbidities may outweigh the generally indolent behavior expected of low-grade dural lymphomas.

**Table 1:** Literature review: cases of dural lymphoma mimicking a subdural hematoma. AE1/AE3: pan-cytokeratin cocktail; Bcl-2: B-cell lymphoma 2 protein; BCL6: B-cell lymphoma 6 protein; CD: cluster of differentiation; C-MYC: cellular myelocytomatosis oncogene; F: female; IgM: immunoglobulin M; Ki-67: proliferation marker (MKI67 antigen); M: male; MALT: mucosa-associated lymphoid tissue; TdT: terminal deoxynucleotidyl transferase; WHO: World Health Organization

Author(s), Year	Age/Sex	Presenting Symptoms	Trauma History	Location	Imaging Findings (CT/MRI)	Treatment/Outcome	Immunophenotype/Diagnosis
Goetz et al., 2002 [10]	64/F	Left hemiparesis, headache	No	Right fronto-parietal	CT: hyperdense, non-enhancing. MRI: isointense T1/T2.	Craniectomy + radiotherapy. No recurrence (3 months).	CD20+, cyclin D1-, Ki-67 6.9%
Benouaich et al., 2003, case 1 [11]	38/F	Not specified	Yes	Left fronto-parietal	CT: hypodense, non-enhancing. MRI: T2 hyperintense, homogeneous enhancement.	Biopsy + radiotherapy. No recurrence (24 months).	CD20+, IgM/IgD-, cyclin D1-
Benouaich et al., 2003, case 2 [11]	45/F	Not specified	Yes	Right temporo-parietal	MRI: isointense T1, homogeneous enhancement.	Biopsy + chemotherapy/radiotherapy. No recurrence (12 months).	CD20+, cyclin D1-
Low and Allen, 2006 [3]	72/F	Grand mal seizure	No	Dural mass (mimicked meningioma)	CT/MRI: meningioma-like extra-axial dural mass.	Surgical excision. Concomitant bone-marrow infiltration; follow-up not specified.	CD20+, follicular pattern, t(14;18)+, grade I
Gocmen et al., 2010 [12]	45/F	Seizures, dysphasia	Yes (6 months prior)	Left fronto-temporal	CT: hypodense, mild enhancement. MRI: T2 hyperintense, homogeneous enhancement.	Craniectomy + biopsy. Follow-up not reported.	CD20+, cyclin D1-, Ki-67 5-10%
Mathon et al., 2013 [13]	59/F	Severe headache, right-sided weakness	No	Left fronto-temporal	CT: hypodense, non-enhancing. MRI: strong post-contrast enhancement.	Biopsy + chemotherapy. Follow-up not reported.	CD20+, Ki-67 95%
Ramnarayan et al., 2013 [7]	59/M	Drowsiness, disorientation, fever	Yes	Left fronto-temporal	CT: hypodense, mild enhancement. MRI: isointense T1/T2.	Craniectomy + steroids + radiotherapy. Died before completing radiotherapy.	CD20/CD79a/CD99/TdT+, Ki-67 8-10%
Ozkul-Wermester et al., 2014 [4]	71/F	Seizures	Yes	Left fronto-parietal	CT: hypodense, non-enhancing. MRI: homogeneous enhancement.	Biopsy + craniectomy + radiotherapy. Follow-up not reported.	Not reported
Low et al., 2014 [14]	62/M	Right upper-limb paresis	No	Left fronto-parietal	CT: hypodense, edge-enhancing. MRI: heterogeneous enhancement.	Craniectomy. Follow-up not reported.	CD20+, CD43+, Bcl-2+, CD10-, S100-
Azevedo et al., 2015 [15]	69/M	Left hemiparesis, headache	No	Right fronto-parietal	CT: hypodense, non-enhancing. MRI: homogeneous enhancement, restricted diffusion.	R-CVP chemotherapy. Follow-up not reported.	Elevated serum IgM (4-26 g/L)
Neeley et al., 2019 [16]	77/F	Transient left facial droop	No	Right fronto-temporal	CT: hyperdense, non-enhancing. MRI: DWI-hyperintense.	Biopsy + radiotherapy. No recurrence (3 months).	Kappa light-chain+, CD43+
Nakaya et al., 2021 [17]	78/F	Headache	No	Right temporo-parietal	MRI: T1-hypointense.	Biopsy only. Follow-up not reported.	CD79a/CD10+, BCL6+, C-MYC+, Ki-67 55%
Thibodeau et al., 2022 [18]	70/F	Dizziness, dysgraphia	No	Left fronto-parietal	CT: hypodense, non-enhancing. MRI: heterogeneous enhancement, restricted diffusion.	Craniectomy + chemotherapy/radiotherapy. No recurrence (12 months).	CD20+, Bcl-2+, Ki-67 80-90%
Hamamoto et al., 2024 [19]	49/F	Transient right upper-limb paresis, dysarthria	No	Left fronto-temporal	CT: hyperdense, non-enhancing. MRI: homogeneous enhancement.	Biopsy + radiotherapy. No recurrence (8 months).	CD20+, CD3+, CD27+, Ki-67 20%
Han et al., 2025 [6]	71/F	Headache, vomiting, papilledema	No	Left fronto-parietal	CT: slightly hyperdense, crescent-shaped. MRI: homogeneous enhancement, DWI-hyperintense, ADC-hypointense.	Biopsy + R-CHOP chemotherapy. Recurrence at 14 months; died at 15 months.	CD20+, CD45+, PAX-5+, Ki-67 20%
Present case, 2026	77/F	Progressive right hemiparesis, hemidysesthesia, memory impairment, headache	No (2 falls; no direct head trauma)	Left fronto-parietal	CT: biconvex, hypodense. MRI: T2 hyperintense, dural enhancement.	Craniotomy + dural resection + chemotherapy. Died 2 months after discharge.	CD20+, Ki-67 30%, interfollicular 95%/follicular 5%, grade I

From an imaging standpoint, the most useful finding for differentiating dural lymphoma from CSDH is homogeneous enhancement of the lesion after gadolinium administration and diffusion restriction (hyperintensity on diffusion-weighted imaging with corresponding hypointensity on the apparent diffusion coefficient map), findings that are not characteristic of an organizing hematoma, in which enhancement - when present - is usually limited to the membrane and is heterogeneous [6]. In the present case, MRI showed frank dural enhancement, a finding that, in retrospect, supported suspicion of a non-hemorrhagic lesion, although the definitive diagnosis could only be established through intraoperative histopathological study.

The differential diagnosis of dural enhancement is broad and includes, in addition to primary dural lymphoma and meningioma, inflammatory and histiocytic entities such as Rosai-Dorfman disease with leptomeningeal involvement, which can present as a dural mass with homogeneous enhancement, radiologically indistinguishable from a meningioma or a lymphoproliferative process, as documented in a Mexican case of leptomeningeal and temporal muscle Rosai-Dorfman disease [8]. This entity, like dural lymphoma, underscores the importance of not basing the definitive diagnosis solely on imaging findings, but always confirming it through histopathological and immunohistochemical study, given that management and prognosis differ substantially between these entities.

Dural involvement is not exclusive to non-Hodgkin lymphoma. Hodgkin lymphoma can also infiltrate the dura mater, most often as a manifestation of metastatic or secondary disease rather than a primary dural process; Elwell et al. described a case of dural infiltration by metastatic Hodgkin's lymphoma [9], illustrating that lymphomatous dural enhancement should remain in the differential diagnosis regardless of lymphoma lineage, particularly in patients with a known or suspected history of Hodgkin lymphoma.

Other relevant differential diagnoses of dural enhancement include idiopathic or secondary hypertrophic pachymeningitis (associated with IgG4-related disease, tuberculosis, or autoimmune disorders), dural metastases, an organized subdural hematoma with vascularized neomembranes, and, less commonly, en plaque meningioma. In all cases, clinical suspicion is decisive in guiding timely histopathological study when imaging findings are discordant with the expected clinical course.

## Conclusions

Primary dural lymphoma, particularly its follicular variant, is an exceptionally rare entity that can clinically and radiologically mimic a CSDH, especially in elderly patients. When findings are discordant - absence of a clear head trauma history, subacute progression of neurological deficit, unexplained cytopenias, and homogeneous dural enhancement after contrast - this differential diagnosis should be considered, along with other infiltrative dural entities such as Rosai-Dorfman disease. Definitive diagnosis always requires histopathological and immunohistochemical confirmation, and prognosis depends primarily on the histological subtype and timely adjuvant oncological treatment.

## References

[1] Ducruet AF, Grobelny BT, Zacharia BE (2012). The surgical management of chronic subdural hematoma. Neurosurg Rev.

[2] Taylor JW, Flanagan EP, O'Neill BP (2013). Primary leptomeningeal lymphoma: International Primary CNS Lymphoma Collaborative Group report. Neurology.

[3] Low I, Allen J (2006). Low-grade follicular lymphoma in the dura: rare mimic of meningioma. Neuropathology.

[4] Ozkul-Wermester O, Guégan-Massardier E, Triquenot-Bagan A, Langlois O, Lefaucheur R, Bourre B (2014). Suspected subdural hematoma. JAMA Neurol.

[5] Iwamoto FM, Abrey LE (2006). Primary dural lymphomas: a review. Neurosurg Focus.

[6] Han Y, Chen B, Zeng M, Liang D, Wang Y, Wang Y (2025). Primary subdural lymphoma mimicking chronic subdural hematoma: a case report and a narrative review of some recent similar cases in the literature. Int J Emerg Med.

[7] Ramnarayan R, Anilkumar T, Nayar R (2013). An unusual extra-axial hypodense lesion mimicking a chronic subdural haematoma. J Neurosci Rural Pract.

[8] Arellanes-Chávez CA, Ordoñez-Solorio LA, Luévano-Flores E (2009). Leptomeningeal and temporal muscle Rosai-Dorfman disease. Arch Neurociencias.

[9] Elwell VA, Carney L, Johns P, Grieve JP (2008). Dural infiltration of metastatic Hodgkin's lymphoma. Br J Neurosurg.

[10] Goetz P, Lafuente J, Revesz T, Galloway M, Dogan A, Kitchen N (2002). Primary low-grade B-cell lymphoma of mucosa-associated lymphoid tissue of the dura mimicking the presentation of an acute subdural hematoma. Case report and review of the literature. J Neurosurg.

[11] Benouaich A, Delord JP, Danjou M (2003). Primary dural lymphoma: a report of two cases with review of the literature. Rev Neurol (Paris).

[12] Gocmen S, Gamsizkan M, Onguru O, Sefali M, Erdogan E (2010). Primary dural lymphoma mimicking a subdural hematoma. J Clin Neurosci.

[13] Mathon B, Nouet A, Villa C (2013). Teaching neuroimages: Burkitt dural lymphoma mimicking a subacute subdural hematoma. Neurology.

[14] Low YY, Lai SH, Ng WH (2014). An unusual presentation of primary malignant B-cell-type dural lymphoma. Singapore Med J.

[15] Azevedo R, Reis F, Brito AB, Vassallo J, Lima CS (2015). Dural lymphoma mimicking subdural haematoma on computerized tomography. Br J Haematol.

[16] Neeley OJ, Al-Hreish KM, Aoun SG (2020). Tumoral mimics of subdural hematomas: case report and review of diagnostic and management strategies in primary B-cell lymphoma of the subdural space. World Neurosurg.

[17] Nakaya A, Ishii K, Nomura S (2021). Primary dural high grade B cell lymphoma mimicking subdural hematoma. Hematol Transfus Cell Ther.

[18] Thibodeau R, Li HK, Babu H, Jafroodifar A, Ramovic M, Hahn SS (2022). Dural lymphoma misdiagnosed as subdural hematoma following head trauma after an episode of syncope. Radiol Case Rep.

[19] Hamamoto R, Kawasaki T, Oda M (2024). Primary extranodal marginal zone mucosa-associated lymphoid tissue-type B-cell lymphoma involving the dura: a case report. Surg Neurol Int.

